# Held still or pressured to receive dental treatment: self-reported histories of children and adolescents treated by non-specialist dentists in Hordaland, Norway

**DOI:** 10.1007/s40368-022-00724-8

**Published:** 2022-06-28

**Authors:** R. S. Aarvik, E. J. Svendsen, M. L. Agdal

**Affiliations:** 1 Institute of Health and Society, Faculty of Medicine, University of Oslo, Forskningsveien 2b, 0373 Oslo, Norway; 2 Oral Health Centre of Expertise in Western Norway, Bergen, Norway; 3 Department of Nursing and Health Promotion, Oslo Metropolitan University, Oslo, Norway; 4 Department of Research, Sunnaas Rehabilitation Hospital, Nesoddtangen, Norway

**Keywords:** Dental treatment, Children and adolescents, Public dental service, Non-specialist dentists, Restraint, Dental fear and anxiety, Trust in dentists

## Abstract

**Aim:**

This study aimed to estimate the prevalence of a self-reported history of restraint in children and adolescents when receiving dental care by non-specialist dentists and to assess differences in dental fear and anxiety (DFA), intra-oral injection fear, and trust in dentists between patients with and without a self-reported history of restraint.

**Methods:**

An electronic cross-sectional survey was distributed to all 9 years old (*n* = 6686) and 17 years old (*n* = 6327) in the Public Dental Service in Hordaland County, Norway, in 2019. For statistical evaluation, we generated descriptive statistics and Mann–Whitney *U* tests.

**Results:**

The response rate ranged between 43.5 and 59.9% for the different questions. The prevalence of a self-reported history of being held still against one’s will during dental treatment and pressured to undergo dental treatment against one’s will was 3.6% and 5.1%, respectively. In general, these patients reported higher DFA, and higher intra-oral injection fear compared with those without such histories of restraint. Patients who had reported being held still against their will during dental treatment had significantly higher distrust in dentists than those who did not report restraint (*p* < 0.001).

**Conclusion:**

To feel pressured to receive dental treatment and to be held still against one’s will overlap with the concepts of psychological and physical restraint. Patients with a self-reported history of restraint recorded significant differences in DFA, intra-oral injection fear, and trust in dentists compared to those who did not report restraint. Future studies should explore the role that restraint may play in relation to a patient’s DFA, intra-oral injection fear, and trust in dentists.

## Introduction

In the past four decades, extensive research has contributed to the understanding of how dental fear and anxiety (DFA) have implications for both adult and paediatric patients’ ability to receive dental care (Armfield et al. 2007; Berggren and Meynert 1984; Seligman et al. 2017). The use of different behavioural approaches to ameliorate the dental situation and help patients overcome dental anxiety has been found to be effective (Berge et al. 2017a, b; Roberts et al. 2010; Seligman et al. 2017). In Norway, approximately 10% of non-specialist dentists educated in the Nordic region reported that they would use restraint if necessary in paediatric dental care of young patients with severe caries (Rønneberg et al. 2017). The use of restraints occurs in situations where the child resists recommended or necessary dental treatment (Aarvik et al. 2021; da Silva et al. 2021; Ilha et al. 2021) and/or perhaps does not fully understand the necessity of dental treatment. In a recent qualitative study, nine Norwegian non-specialist dentists reported the occasional use of restraints to complete necessary dental treatment, despite being uncertain of possible harmful consequences for the child (Aarvik et al. 2021).

The use of restraint can be considered a necessary approach when other behavioural and/or pharmacological techniques are not available and dental treatment needs are both extensive and urgent (Aarvik et al. 2021; Ilha et al. 2021; da Silva et al. 2021; Marty et al. 2020; Rønneberg et al. 2017). Dental health services are required to provide treatment while respecting the integrity of each individual and obtain informed consent (Lovdata 1999). In dental literature, terms that are used to describe treatment without the patient’s will and acceptance are ‘restraint’, ‘protective stabilisation (against ones will)’, ‘active immobilisation’, ‘passive immobilisation’, and ‘clinical holding’ (Aarvik et al. 2021; American Academy of Pediatric Dentistry 2021; Armfield and Heaton 2013; British Society of Paediatric Dentistry 2016; da Silva et al. 2021; Ilha et al. 2021; Vargas et al. 2007). Being held down while being fearful or resisting the treatment is probably not compatible with a feeling of control in the situation. In our study, we therefore opted to use the broader term ‘restraint’ to encompass the different techniques that may be used by dental health personnel or parents/caregivers to proceed with dental treatment against a child’s will.

A systematic review by Zhou et al. indicate that dental staff behaviours such as coercion, coaxing, putdowns, stopping treatment, and holding and restraining are associated with fear-related behaviours in children (Zhou et al. 2011). These findings relate to studies by Weinstein et al. (1982, 1983) observing 3- to 5-year-old children during dental treatment visits that included local anaesthetic administration, and follow-up observations were not undertaken. Dental fear and dental anxiety are distinctly different. Fear is an adaptive reaction to fearful stimuli, whereas anxiety is not. Klingberg and Broberg (2007) defined DFA as ‘strong negative feelings associated with dental treatment among children and adolescents’. We adopted the term ‘DFA’ to describe all levels of dental fear and anxiety given that the terms ‘fear’ and ‘anxiety’ are often used interchangeably by clinicians. Approximately 5–20% of children and adolescents experience high DFA or high fear of intra-oral injections, with the variation attributed to differences in study populations and study design (Berge et al. 2016; Klingberg and Broberg 2007; Stenebrand et al. 2013). In the adult population, DFA is associated with reduced oral health (Hakeberg et al. 1993) and quality of life (Berggren 1993). For many, DFA and intra-oral injection fear can result in dental avoidance (Armfield et al. 2007; Berge et al. 2016; Berggren and Meynert 1984; Seligman et al. 2017).

The aetiology of DFA is considered to consist of a complex interplay of cognitive, behavioural, and contextual factors, and it has been proposed that a common factor in the development of DFA is a direct conditioning experience—most frequently a painful or traumatic dental experience (Seligman et al. 2017; Skaret et al. 1999). Knowledge concerning children’s perception of restraint has seldom been assessed, but may be valuable and important in informing best clinical practice. To the best of our knowledge, no study has investigated the self-reported histories of the use of restraints during dental treatment in children and adolescents. Since this study is novel in its focus on restraint, it was necessary to have an explorative approach to gain knowledge that might guide to the development of prospective studies in the future. Therefore, this study aimed to estimate the prevalence of a self-reported history of restraint in children and adolescents when receiving dental care by non-specialist dentists and to assess differences in dental fear and anxiety (DFA), intra-oral injection fear, and trust in dentists between patients with and without a self-reported history of restraint.

## Materials and methods

We distributed an electronic cross-sectional questionnaire directly to all 17-year-old adolescents and addressed to all 9-year-old children via their parents’ phone number in the Public Dental Service (PDS) in the county of Hordaland, Norway. The age group “9-year-olds” were considered old enough to have experience with dental treatment and to be able to report on their subjective experiences. An age close to the potential self-reported restraint was assumed to lower the risk of recall bias. The PDS in Norway is responsible for individually adapted, free-of-charge follow-up of oral health of children and adolescents aged up to 18 years (Lovdata 1983). The age group “17-year-olds” were addressed to include persons who still were patients in the PDS and could report on their accumulated experiences in the PDS. Hordaland County, which includes Norway's second largest city (Bergen), was in 2019 the third most populated county in Norway (Statistics Norway 2019a). The county is mostly rural and sparsely populated outside of the Bergen metropolitan area, which reflects the country. The median household income is similar to the median national household income (Statistics Norway 2019b). Thus, Hordaland can be regarded representative for epidemiological research in Norway. Most dentists in the Norwegian PDS are general dentists, and of all dentists, approximately 1% (47) are specialists in paediatric dentistry (Statistics Norway 2019c).

### Data collection

Data were collected from October to December 2019, and the survey was distributed using the PDS text message function in the journal system. The 17 years old received the invitation as a text message on their own phones, whereas the 9 years old received it on their parents’ phones specified with the name of the child. By legislation, all patients below the age of 16 years are to be contacted through their parents in the Norwegian healthcare system. The parents were informed to assist the child, and the message specified that the study sought to examine the child’s subjective experiences. Given the anonymous design of the study, we sent one invitation and three reminders (at 2, 6, and 8 weeks) to all individuals. The text messages provided a link to the survey (estimated to take 10 min), which also obtained informed consent to participate in the study. The survey was written in Norwegian. One iPad in each age group was raffled as an incentive for participation.

### Survey

This paper examined the following elements obtained in the cross-sectional survey: demographic details (sex and age), self-reported history of restraint at the dental clinic, potential fear of dental treatment and intra-oral injections, and trust in dentists.

To our knowledge, no psychometric instruments about self-reported histories of restraint for this group of patients have been developed. As such, we developed seven items based on earlier research and the definition of restraint (Bray et al. 2015; Svendsen et al. 2015; Kangasniemi et al. 2014). These items were thoroughly discussed in the research group, with psychologists and specialists in paediatric dentistry, and thereafter tested on the respective age groups. Comments from the test group showed that the developed questions were easy to understand and answer. Research on restraint is context dependent, and passive immobilisation, such as via a papoose board or Pedi wrap, is not used in the Norwegian PDS. Therefore, passive immobilisation was not addressed in the survey. Being held still against the one’s will (physical restraint) was measured by the item, ‘Have you experienced being held still against your will during dental treatment?’ (*yes*, *no*, or *do not know*). Respondents who answered *yes* were asked the following questions: ‘Have you experienced being physically held still against your will during dental treatment several times?’ (*yes*, *no*, and *do not know*), ‘Approximately how old were you when/the first time you experienced being physically held still against your will during dental treatment?’, ‘Approximately, how old were you the last time you experienced being physically held still against your will during dental treatment?’ (age), and ‘In what/which situation(s) were you being physically held still during dental treatment?’. Situational descriptions of when physical restraint was experienced are presented in Table 2 under “Results”. Then, the question ‘Have you felt pressured to receive dental treatment in such a way that you could not say no?’ (*no degree*, *low degree*, *neither high nor low*, *high degree*, or *very high degree*) followed. The item ‘Have you wanted to escape from the dental treatment situation?’ served as follow-up question.

To measure DFA, we used the psychometric instrument Children’s Fear Survey Schedule-Dental subscale (CFSS-DS) (Cuthbert and Melamed 1982), which consists of 15 questions related to different aspects of dental treatment. Each item is scored from 1 (*not afraid at all*) to 5 (*very afraid*) with a sum score ranging from 15 to 75. The CFSS-DS is a widely used instrument for measuring DFA, among others in Norwegian and Swedish children and adolescents (Berge et al. 2016; Gustafsson et al. 2010). This study used the self-report version with a suggested cut-off score > 38 to indicate high DFA (Gustafsson et al. 2010). To measure intra-oral injection fear, the Intra-Oral Injection Fear-scale (IOIF-s) (Berge et al. 2017a, b) was used. This 12-item questionnaire has been validated in Norway for children aged from 10 to 16 years, with items scored from 1 (*not afraid at all*) to 5 (*very afraid*); sum scores range from 12 to 60. A cut-off score of > 38 indicates high fear of intra-oral injections (Berge et al. 2017a, b).

We used eight single items (presented in Table 4) based on the Getz Dental Beliefs Survey (DBS) (Kvale et al. 1997) to measure patients’ trust in dentists. The questions cover different situations, feelings, and thoughts that may occur during dental treatment and are rated on a Likert scale from 1 to 5 (*never*, *one or two times*, *a few times*, *often*, or *almost always*). Only parts of the instrument were used to shorten the survey’s length and thereby reduce the risk of dropouts, with the knowledge that only some aspects of trust in dentists were measured. Therefore, no sum score is presented. Since the DBS is not validated in children, and 17 years old can be considered adults, the items are analysed and presented separately for the different age groups.

### Data and statistical analysis

All participants who answered the survey were included in the analysis. The dichotomised variables followed this pattern: items with the response alternatives *yes*/*no*/*do not know* were coded 0 for *no*/*do not know* and 1 for *yes*, and the five-point items were coded 0 for *not at all*/*low degree*/*neither high nor low* and 1 for *high degree*/*very high degree*.

Statistical analyses were performed using IBM SPSS Statistics for Windows, version 26.0 (Armonk, NY, USA). Descriptive statistics were generated using ‘Frequencies’. We used Mann–Whitney *U* tests to compare group differences. The level of statistical significance was set at *p* < 0.05. The option ‘exclude cases pairwise’ was chosen in all analyses with missing data, indicating that the respective cases were excluded only if they had missing data required for the specific analysis.

### Ethical approvals

The Norwegian Centre for Research Data (#783349/2019) and County Dental Officer in Hordaland approved this study. Additionally, the content, the recruitment procedure, and length of the survey were discussed with psychologists at the Centre for Odontophobia (Oral Health Centre of Expertise in Western Norway) in Bergen. The survey was considered unlikely to have negative consequences for the respondents.

## Results

In total, 13,013 adolescents (aged 17 years, *n* = 6327) and children (aged 9 years, *n* = 6686), assisted by their parent(s), were invited to participate in this study. The total response rate ranged from 43.8 to 59.9% for the different questions in the survey. Among the participants, 50.0% identified as *boys* (*n* = 3844), 49.8% as *girls* (*n* = 3832), and 0.2% as *they* (*n* = 12). Table 1 presents the descriptive statistics for the age groups.

**Table 1:** Descriptive statistics for individuals aged 17 (born 2002) and 9 years (born 2010)

Item	*n* (%)
Year of birth	
2002	3305 (52.2)
2010	4383 (65.6)
Self-reported physical restraint	
17 years old	75 (2.9)
9 years old	130 (4.2)
Self-reported being pressured to accept dental treatment	
17 years old	159 (6.0)
9 years old	137 (4.3)
High DFA (CFSS-DS > 38)	
17 years old	162 (5.5)
9 years old	277 (8.1)
High intra-oral injection fear (IOIF-s > 38)	
17 years old	339 (13.2)
9 years old	493 (15.9)

### Prevalence of a reported history of restraint

The prevalence of a self-reported history of being held still against one’s will (physical restraint) during dental treatment was 2.9% (*n* = 75) for 17 years old and 4.2% (*n* = 130) for 9 years old. In total, 3.6% (*n* = 205) of patients reported a history of physical restraint. Of them 43.6% (*n* = 89) had reported the use of physical restraint several times, and 29.0% (*n* = 58) reported the use of physical restraint under conscious sedation. Physical restraint was reported by both age groups to have occurred most often when the child was 5–9 years old. Table 2 shows the distribution of the different dental situations where the respondents reported physical restraint. In total, the reported prevalence of having felt pressured to receive dental treatment in such a way that one could not say no, was 5.1% (*n* = 296).

**Table 2:** In descending order, these are the situational descriptions of when physical restraint was reported

Situational descriptions of when physical restraint was reported	*n* (%)
The dentist said I needed dental treatment	135 (67.2)
When I tried to escape from the dental chair	101 (50.1)
When I could not sit still in the dental chair	86 (43.2)
I had toothache and contacted the dental clinic for help	72 (35.6)
Other situation not specified	66 (33.2)
When I had been administered sedative medication	58 (29.0)
I hit one tooth/several teeth and needed dental treatment	47 (23.5)

The column ‘*n* (%)’ represents the *yes* responses for each item

### Dentistry-related fear and restraint

Mann–Whitney *U* tests indicated that participants who reported the use of physical restraint, regardless of age, had significantly higher DFA (CFSS-DS) and intra-oral injection fear (IOIF-s) compared with participants who did not report the use of physical restraint (Table 3). Table 3 gives the results for self-reports of physical restraint and for being pressured to receive dental treatment in such a way that one could not say no.

**Table 3:** Mann–Whitney *U* tests comparing CFSS-DS and IOIF-s scores between patients who reported physical restraint and being pressured against one’s will with patients who reported no physical restraint and not being pressured against one’s will

	Held still against one’s will during dental treatment		Not held still against one’s will during dental treatment		Statistics
	*n*	Median, mean (SD)	*n*	Median, mean (SD)	Mann–Whitney *U* test
CFSS-DS					
17 years old	75	28.0, 29.0 (10.3)	2485	20.0, 22.6 (7.9)	*U* = 129,097, *z* = 5.71, * p* < **0.001**
9 years old	130	34.0, 35.3 (13.1)	2971	24.0, 25.3 (7.7)	*U* = 284,437, *z* = 9.15, * p* < **0.001**
Total	205	31.0, 33.0 (12.5)	5456	22.0, 24.10 (7.9)	*U* = 806,999, *z* = 10.78, * p* < **0.001**
IOIF-s					
17 years old	75	32.0, 31.6 (9.8)	2485	29.0, 29.2 (8.8)	*U* = 109,836, *z* = 2.65, ***p* = 0.008**
9 years old	130	29.0, 30.1 (11.5)	2972	29.0, 26.8 (12.6)	*U* = 214,736, *z* = 2.16, ***p* = 0.031**
Total	205	31.0, 30.7 (10.9)	5457	29.0, 27.9 (11.1)	*U* = 628,060, *z* = 2.99, ***p* = 0.003**

	Pressured against one’s will during dental treatment		Not pressured against one’s will during dental treatment		Statistics
	*n*	Median, mean (SD)	*n*	Median, mean (SD)	Mann–Whitney *U* test
CFSS-DS					
17 years old	159	25.0, 28.6 (12.7)	2478	20.0, 22.5 (7.5)	*U* = 246,211, *z* = 5.30, ***p* < 0.001**
9 years old	137	34.0, 34.9 (12.8)	3035	24.0, 25.4 (7.8)	*U* = 3,052,222, * z* = 9.29, ***p* < 0.001**
Total	296	29.0, 31.5 (13.1)	5513	22.0, 24.1 (7.8)	*U* = 1,081,223, * z* = 9.45, ***p* < 0.001**
IOIF-s					
17 years old	149	27.0, 27.8 (10.9)	2414	29.0, 29.3 (8.7)	*U* = 165,233, * z* = -1.67, *p* < 0.095
9 years old	131	29.0, 29.5 (11.7)	2974	29.0, 26.8 (12.6)	*U* = 211,268, * z* = 1.64, *p* < 0.101
Total	280	28.0, 28.6 (11.3)	5388	29.0, 27.9 (11.1)	*U* = 758,095, * z* = 0.14, *p* < 0.887

**Fig. 1:**
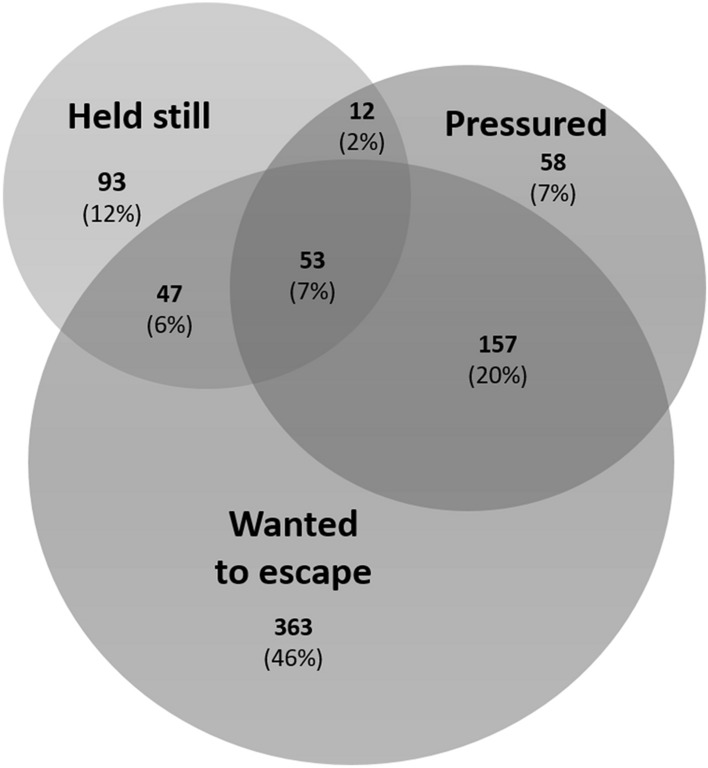
Venn diagram showing the overlap between a self-reported history of physical restraint, having felt pressured to receive dental treatment in such a way that one could not say no, and having wanted to escape from the dental situation. This figure represents respondents who answered all three questions (*n* = 783)

Figure 1 presents a Venn diagram that demonstrates the overlap between a history of being held still (physical restraint), having felt pressured to receive dental treatment in such a way that one could not say no, and having wanted to escape from dental treatment.

### Trust in dentists and restraint

Mann–Whitney *U* tests indicated that the group that reported physical restraint had significantly higher scores for all items measuring distrust in dentists compared with the group that did not report a history of physical restraint during treatment (Table 4).

**Table 4:** Comparison of the frequency of dentist distrust measured by eight items of the Getz Dental Belief Survey among patients with and without a self-reported history of physical restraint during dental treatment

Items from Dental Belief Survey	Held still against one’s will during dental treatment		Not held still against one’s will during dental treatment		Statistics
	*n*	Median, mean (SD)	*n*	Median, mean (SD)	Mann–Whitney *U* test
1					
17 years old	75	2.00, 2.35 (1.30)	2485	1.00, 1.72 (1.27)	*U* = 122,217, *z* = 5.58, ***p* < 0.001**
9 years old	130	2.00, 2.28 (1.38)	2972	1.00, 1.60 (1.18)	*U* = 253,192, *z* = 7.65, ***p* < 0.001**
Total	205	2.00, 2.31 (1.35)	5457	1.00, 1.66 (1.22)	*U* = 730,955, *z* = 9.29, ***p* < 0.001**
2					
17 years old	75	2.00, 2.65 (1.40)	2485	1.00, 1.60 (1.02)	*U* = 133,897, *z* = 7.61, ***p* < 0.001**
9 years old	130	2.00, 2.34 (1.37)	2972	1.00, 1.33 (0.82)	*U* = 280,165, *z* = 12.58, ***p* < 0.001**
Total	205	2.00,2.45 (1.39)	5457	1.00,1.45 (0.93)	*U* = 800,255, *z* = 13.52, ***p* < 0.001**
3					
17 years old	75	2.00, 2.31 (1.43)	2485	1.00, 1.55 (1.13)	*U* = 123,402, *z* = 6.32, ***p* < 0.001**
9 years old	130	2.00, 2.36 (1.48)	2972	1.00, 1.48 (1.20)	*U* = 264,204, *z* = 9.80, ***p* < 0.001**
Total	205	2.00, 2.34 (1.46)	5457	1.00,1.51 (1.11)	*U* = 754,575, *z* = 11.48, ***p* < 0.001**
4					
17 years old	75	1.00, 1.85 (1.12)	2485	1.00, 1.20 (0.67)	*U* = 126,732, *z* = 9.53, ***p* < 0.001**
9 years old	130	1.00, 1.78 (1.23)	2972	1.00, 1.17 (0.59)	*U* = 248,197, *z* = 10.22, ***p* < 0.001**
Total	205	1.00, 1.80 (1.19)	5457	1.00,1.18 (0.63)	*U* = 733,522, *z* = 13.84, ***p* < 0.001**
5					
17 years old	75	3.00, 2.75 (1.46)	2485	1.00, 1.61 (1.06)	*U* = 135,861, *z* = 8.05, ***p* < 0.001**
9 years old	130	3.00, 2.81 (1.46)	2972	1.00, 1.48 (0.91)	*U* = 294,521, *z* = 12.55, ***p* < 0.001**
Total	205	3.00, 2.79 (1.46)	5457	1.00,1.54 (0.98)	*U* = 836,574, *z* = 14.65, ***p* < 0.001**
6					
17 years old	75	2.00, 2.27 (1.31)	2485	1.00, 1.58 (1.18)	*U* = 123,920, *z* = 6.35, ***p* < 0.001**
9 years old	130	2.00, 2.32 (1.43)	2972	1.00, 1.59 (1.24)	*U* = 258,117, *z* = 8.61, ***p* < 0.001**
Total	205	2.00,2.30 (1.38)	5457	1.00,1.58 (1.21)	*U* = 745,690, *z* = 10.66, ***p* < 0.001**
7					
17 years old	75	2.00, 2.17 (1.17)	2485	1.00, 1.24 (0.69)	*U* = 139,786, *z* = 11.89, ***p* < 0.001**
9 years old	130	1.00, 1.90 (1.16)	2972	1.00, 1.17 (0.62)	*U* = 269,391, *z* = 13.68, ***p* < 0.001**
Total	205	2.00, 2.00 (1.17)	5457	1.00,1.20 (0.65)	*U* = 799,668, *z* = 17.80, ***p* < 0.001**
8					
17 years old	75	2.00, 2.28 (1.24)	2485	1.00, 1.30 (0.78)	*U* = 138,526, *z* = 10.70, ***p* < 0.001**
9 years old	130	1.00, 2.03 (1.32)	2972	1.00, 1.25 (0.73)	*U* = 264,109, *z* = 11.52, ***p* < 0.001**
Total	205	2.00, 2.12 (1.29)	5457	1.00,1.27 (0.76)	*U* = 787,339, *z* = 15.44, ***p* < 0.001**

A low score indicates high trust

Bold values denote statistical significance at the *p* < 0.05 level

Items from Dental Beliefs Survey accordingly numbers in table: 1. Dentists do not seem to care that patients sometimes need a rest, 2. Dentists focus too much on getting the job done and not enough on the patient’s comfort, 3. I am concerned that dentists will not take my worries (fears) about dentistry seriously, 4. I am concerned that dentists will put me down (make light of my fears), 5. Once I am in the chair I feel helpless (that things are out of my control), 6. If I were to indicate that it hurts, I think that the dentist would be reluctant to stop and try to correct the problem, 7. I have had dentists not believe me when I said I felt pain, and 8. I am concerned that the dentist will do what he want and not really listen to me, while I am in the chair

## Discussion

The study identified some 17-year-old adolescents and 9-year-old children who reported the use of physical restraint while undergoing dental treatment. These participants had significantly higher dentistry-related fear and tended to trust dentists significantly less compared with those without a history of restraint during dental treatment. To estimate a prevalence on restraint will vary depending on who is being asked (patient, parent, dental health personnel) and how and what type of instruments are used in the data collection. To the best of our knowledge, the prevalence of a self-reported history of restraint during dental treatment of a child or adolescent in a public dental service has never been examined, and our study provides new knowledge on young patients’ reports of restraint in this setting. Although there are methodological challenges in including children in research, to involve the child’s voice is considered valuable by the United Nation on the Rights of the Child chapter 12 (UN General Assembly 1989), and can provide a unique perspective on what concerns children (James 2007).

One central finding was that some children and adolescents have felt pressured to accept dental treatment. This phenomenon is not identified or conceptualised in guidelines in the field of paediatric dentistry. Interestingly, even though there is no clear consensus, other health care fields have suggested concepts such as physical, psychological, and pharmacological restraint (also called chemical), such as paediatric nurses’ perceptions of the use of restraint in somatic paediatric care (Kangasniemi et al. 2014), emergency paediatric psychiatric evaluation (Dorfman and Kastner 2004), and adult psychiatry (Negroni 2017). Physical restraint involves the use of physical techniques to prevent the child from moving, such as parents and dental health personnel holding the child’s arms, head, and/or legs still when the child resists by moving and/or verbally giving signs of disapproval. Psychological restraint involves verbally or non-verbally pressuring a child to undergo treatment against their will, giving the child the feeling that refusal is not an option. When a medication is administered to sedate an agitated patient to prevent harmful behaviour to the patient or to others, it can be considered as pharmacological or chemical restraint. Although the terminology and concept of restraint is mostly used in other fields of health care, highlighting aspects of less child-friendly practices is of value also in paediatric dentistry and research. Since the question “Have you felt pressured to receive dental treatment in such a way that you could not say no?” overlaps the concept psychological restraint, we have adopted that concept in this study.

The prevalence of self-reported physical restraint was higher in the 9-year age group, although the 17-year age group, who reported from a longer period of life, probably had more need for urgent dental treatment. The retrospective design of the study implies a risk of recall bias (Bowling 2014). Older participants may have habituated to the dental situation over time, and memories of events may have faded, or they have displaced prior events. Given that the child’s right to participate in decision-making has been on the agenda of society and healthcare services for some time (Coyne 2008), a reduction in the self-reported history of physical restraint between the two groups was expected. In a recent qualitative study, Norwegian public dentists reported that the use of physical restraint is the most common in the age group 5–9 years and when the child is sedated (Aarvik et al. 2021).

Self-reports of psychological restraint were more prevalent in the 17-year-old group. With increased maturity, 17-year-old adolescents may to a larger degree better understand the need for dental treatment than 9-year-old children (Bee and Boyd 2007), and psychological restraint involves verbally or non-verbally pressuring a child to accept treatment against their will. For some participants, the answer to the question on psychological restraint can be rooted in an accurate understanding of the need for dental treatment, whereas the experience of others can be rooted in a situation where they felt pressured by the dental health personnel and/or their parents/caregivers to undergo dental treatment. In this study, almost 75% of the patients who reported a history of psychological restraint had an urge to escape from dental treatment (Fig. 1). One of the diagnostic criteria for a specific phobia, such as dental phobia, is described in the Diagnostic and Statistical Manual of Mental Disorders, Fifth Edition, (American Psychiatric Association 2013) as “the phobic object or situation is actively avoided or endured with intense fear or anxiety.” Armfield (2010) argued that questionnaires on DFA should incorporate elements of the diagnostic criteria of specific phobia. Therefore, we included the question about escaping treatment. Since children are commonly accompanied by their parents or caregivers, they rarely avoid dental visits, unlike the case among adolescents and adults.

In the present study, the three most common reasons for experiencing physical restraint were when the dentist stated that dental treatment was necessary and when the patient tried to escape or could not sit still. The use of restraint when treatments are considered necessary has been identified in both dental (Aarvik et al. 2021) and health service literature (Kangasniemi et al. 2014). Since many children and adolescents reported that they had experienced physical restraint when they somehow physically resisted, it is likely that the dentist might describe them as having behavioural management problems (BMP) (Klingberg et al. 1994).

Many of the participants with a self-reported history of restraint reported the use of restraint several times. Owing to the retrospective design of our study, we could not obtain information on the participants’ degree of DFA before the use of restraint. We can hypothesise that multiple instances of restraint might explain some of the difference in DFA between those who reported a history of restraint and those without a history of restraint; multiple negative events tend to increase the risk of developing DFA (Skaret et al. 1999). Skaret et al. (1999) noted that 18-year-old students who reported more than one previous episode of pain during attendances at the PDS in Norway were ten times more likely to report high dental anxiety than the rest of the group. On the other hand, DFA may interfere with a patient’s perception of restraint and self-reports of restraint may be over-reported in patients with DFA. In this study, 29.0% of children and adolescents who reported physical restraint had their dental treatment provided under conscious sedation. One would expect many of those children to have DFA preoperatively as DFA/BMP would be the likely reason for scheduling treatment under sedation. The development of DFA or dental phobia is a complex interaction of multiple factors, such as general and psychological health, poor oral health, painful dental treatment and Molar Incisor Hypomineralisation, or other oral conditions that might involve painful dental treatment, and environment (Seligman et al. 2017; Skaret et al. 1999). A limitation of our study is its cross-sectional design which means that causality cannot be inferred from its results. Therefore, it is impossible from our study to determine if DFA caused the need to use restraint or if the use of restraint caused DFA. It is acknowledged that DFA has a multifactorial aetiology, and the authors of this paper recommend that the role of restraint as a factor in the development of DFA should be explored in future prospective studies.

In the UK, the use of restraint (clinical holding) when providing dental care for children is limited to specialists in paediatric dentistry or in special care dentistry who have had formal training in such advanced behaviour guidance procedures (British Society of Paediatric Dentistry 2016). Contrary, in the US, the use of restraint (protective stabilisation) during dental care is “considered within an overall behaviour guidance plan that promotes a positive dental attitude and quality of care” (American Academy of Pediatric Dentistry 2021). In Norway, new national guidelines for dentists treating patients from 0 to 20 years were published on the 31st of March 2022 (Norwegian Directorate of Health 2022). The guideline on the use of restraint recommends that restraint shall be a last-resort treatment method only for dental treatment that cannot be postponed, after consultation with a specialist in paediatric dentistry if necessary. Dentists and paediatric dentists educated in Norway are not trained in administering restraint.

The response rate to the survey was lower in the 17-year age group than in the 9-year age group. It is known that avoidance behaviour to dental triggers is prevalent among individuals with high DFA. However, in a national epidemiological survey of oral health in Australia (Armfield et al. 2009), the response rate of individuals with dental fear and phobia was not appreciably lower than that of other individuals in the survey. Nonetheless, in our study, those 17 years old with severe DFA who avoid dental situations, such as hearing and speaking about dentists, may not have opened the text message from ‘the dentist’. Given that DFA in general was higher for those with a self-reported history of restraint, our study design may have resulted in us missing some of the most anxious patients who might have a history of restraint.

Patients with a self-reported history of restraint had significantly less trust in dentists than patients with no history of restraint. Strøm et al. identified that 6% of a strategic sample of 18 years old in Norway have a high distrust of dentists (Strøm et al. 2020). They found that the majority of individuals with distrust also have a high DFA (Strøm et al. 2020). In adults, key elements for successful management of DFA using cognitive behavioural therapy (CBT) is to emphasise the patients’ experience of control in the dental situation and to establish a trustful patient–dentist relationship (Haukebø et al. 2007). CBT has been shown as an effective treatment method for fearful paediatric dental patients, such as for children with intra-oral injection phobia (Berge et al. 2017b). Providing the patient with a sense of control and predictability in the situation is difficult when the patient is restrained. When patients experience that both trust and control are put aside to pursue necessary dental treatments, their terms for the future achievement of good oral health may be challenged.

### Limitations

The survey was carried out in Hordaland County which is considered representative for Norway. The response rate is considered adequate for electronic surveys (McLeod et al. 2013), but the number of non-responders must be considered when interpreting the results. The survey design had a theoretical possibility of being taken multiple times. For ethical considerations, the non-responders were unknown to the authors. Therefore, we could not obtain information on the reasons for non-participation. However, the results on the prevalence of DFA (CFSS-DS) and intra-oral injection fear (IOIF-s) did not differ substantially from a representative study in a similar population with a high response rate (Berge et al. 2016). One weakness of our study is the application of seven non-validated items regarding restraint. Nevertheless, in our opinion, these questions contributed to this underexplored area of research. In future, validated questionnaires regarding restraints should be developed.

The intention of this study was to gain knowledge on children’s and adolescents’ experiences, but it should be acknowledged that the retrospective measure of restraint might include recall bias. Owing to the electronic design of the survey, we could not determine if the children’s answers were entirely self-reports or the degree to which they were mixtures of self-and proxy reports. Parents may in some cases remember situations where restraint has been used that the child has no memory of. In other situations, the child’s subjective experience of restraint may not be apparent for the parents. How proxy reports affect the results of the experiences of restraint is unknown. Regarding DFA, proxy reports have discrepancies with self-reports where parents rate their child’s fear higher than the child would (Gustafsson et al. 2010; Klingberg and Broberg 2007). Thus, whether other cut-off scores on the CFSS-DS and IOIF-s should have been used for the 9-year-old patients can be discussed.

In the Norwegian PDS, paediatric patients are mainly followed up by non-specialist dentists and dental hygienists, and only referred to specialists in paediatric dentistry in special cases. Therefore, we cannot know for sure that the participants’ reports do not include specialist treatment, but have chosen to write non-specialist dentists as that represents most paediatric dental care in Norway.

This study did not include questions about nationality, and as such, we could not confirm the degree to which we obtained responses from participants with a native language other than Norwegian. In the Scandinavian setting, immigrants often have poorer oral health than the general population (Stecksén-Blicks et al. 2014). Therefore, they undergo more dental treatment with possible restraint situations. Furthermore, we do not know to which degree the sample include participants with special health care needs. Other possible reasons for non-participation may be survey fatigue, poor timing, and the assumption that the survey was spam.

## Conclusion

This is the first study to report on the prevalence of self-reported history of restraint during dental treatment among children and adolescents. A small proportion of 17- and 9-year-old patients in Hordaland, Norway, self-report history of restraint during paediatric dental treatment. In general, patients with self-reported history of restraint during dental treatment have higher dentistry-related fear and higher distrust in dentists compared without such history. As thoughts and feelings are activated during dental treatment, scholars studying clinical practice should acknowledge patients’ experience. How restraint may play a role in patients’ DFA, intra-oral injection fear, and trust in dentists should be explored in future studies.

## Data Availability

The data that support the findings of this study are available from the corresponding author upon reasonable request.
